# Mitral valve repair

**DOI:** 10.12688/f1000research.7521.1

**Published:** 2016-06-10

**Authors:** Alberto Pozzoli, Michele De Bonis, Ottavio Alfieri

**Affiliations:** 1 Department of Heart Surgery, Vita-Salute University of Milan, Milan, Italy

**Keywords:** Mitral valve, Mitral regurgitation, Transesophageal echocardiography, Percutaneous intervention

## Abstract

Mitral regurgitation (MR) is the most common valvular heart disease in the Western world. The MR can be either organic (mainly degenerative in Western countries) or functional (secondary to left ventricular remodeling in the context of ischemic or idiopathic dilated cardiomyopathy). Degenerative and functional MR are completely different disease entities that pose specific decision-making problems and require different management. The natural history of severe degenerative MR is clearly unfavorable. However, timely and effective correction of degenerative MR is associated with a normalization of life expectancy. By contrast, the prognostic impact of the correction of functional MR is still debated and controversial. In this review, we discuss the optimal treatment of both degenerative and functional MR, taking into account current surgical and percutaneous options. In addition, since a clear understanding of the etiology and mechanisms of valvular dysfunction is important to guide the timing and choice of treatment, the role of the heart team and of echo imaging in the management of MR is addressed as well.

## Introduction

Mitral regurgitation (MR) is the most frequent clinically recognizable valvular heart disease in the Western world ^1^. MR can be divided into organic MR, resulting from primary anatomical changes of the leaflets and subvalvular apparatus, or functional (secondary) MR, which is a consequence of annular dilatation and geometrical distortion of the subvalvular apparatus secondary to left ventricular (LV) remodeling and dyssynchrony, most usually associated with cardiomyopathy or coronary artery disease (mitral valve [MV] is morphologically normal). Organic and functional MR are different entities with regard to pathophysiology, prognosis, diagnosis, and management and will be discussed separately in this review. The most common etiology of organic MR in industrialized countries is degenerative MV disease, as a result either of myxomatous degeneration or of fibroelastic deficiency of the leaflets, leading to MV prolapse. Less common is organic MR due to rheumatic heart disease (prevalent in developing countries) and congenital MV anomalies ^2^. Functional MR worsens the prognosis of patients with dilated cardiomyopathy ^3, 4^. Ischemic MR is a subcategory of functional MR in which LV dysfunction is the consequence of a previous myocardial infarction. The natural history of severe MR is clearly unfavorable, leading to LV failure, pulmonary hypertension, atrial fibrillation (AF), stroke, and death ^5^. Appropriate and timely correction of degenerative MR, however, has a highly beneficial impact on the prognosis of patients and can even be associated with a life expectancy and a quality of life similar to those of the general population. For functional MR, surgery is challenging and outcomes are inferior to those of degenerative MR, and the indications and choice of technique are not supported by robust evidence ^6^.

Furthermore, in recent years, a variety of approaches for the percutaneous treatment of MR have emerged. The most widely adopted has been the edge-to-edge (EE) procedure investigated in large registries and small randomized trials. Meanwhile, numerous alternative technologies are in development.

## The heart team

A multidisciplinary heart team (interventional cardiologists, cardiac surgeons, anesthesiologists, imaging, and heart failure specialists) should evaluate the advantages and the drawbacks of surgical, percutaneous, and conservative approaches in all high-risk subjects with MR, assessing the risk ratio due to the presence of relevant comorbidities. The team should assess the possible futility of intervention in very high-risk patients, in whom conservative management could be more appropriate. Risk assessment is fundamental to decision-making since percutaneous MV intervention should currently be reserved for high-risk or inoperable patients. Definitions of ‘high surgical risk’ and the ‘inoperable patient’ remain elusive and significantly influenced by surgeon and center experience. Established risk scores—for example, Society of Thoracic Surgeons (STS) and EuroScore II—should be used in conjunction with other factors (e.g. frailty, porcelain aorta, and so on) as recommended by the MVARC (Mitral Valve Academic Research Consortium) consensus documents ^7, 8^. As heart valve teams generate the most beneficial treatments, it becomes fundamental to define the “centers of excellence” in MV repair. Criteria should include MV surgery volume requirements (center and surgeon), appropriate periprocedural imaging skills, and a willingness to provide patients and referring doctors with the data regarding outcomes based on the experience of the institution (including repair rates, mortality rates, stroke rates, and the likelihood of durability of the repair) ^9^. A tailored approach for individual patients remains appropriate in the absence of guidelines for the conduct of heart team activity.

## Imaging assessment

**Figure 1.**
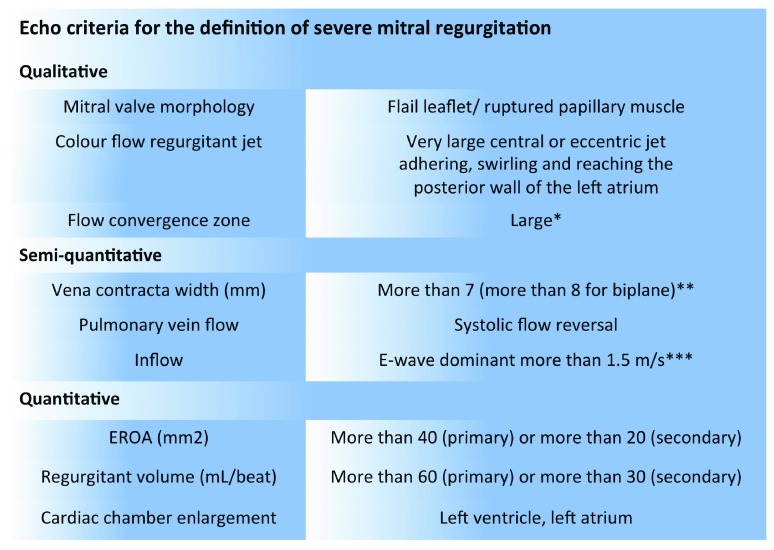
Echo criteria for the definition of severe mitral regurgitation. *Nyquist limit 50–60 cm/s. **Average between apical four- and two-chamber views. ***In the absence of mitral stenosis or other causes of elevated left atrial pressure. EROA, effective regurgitant orifice area.

**Figure 2.**
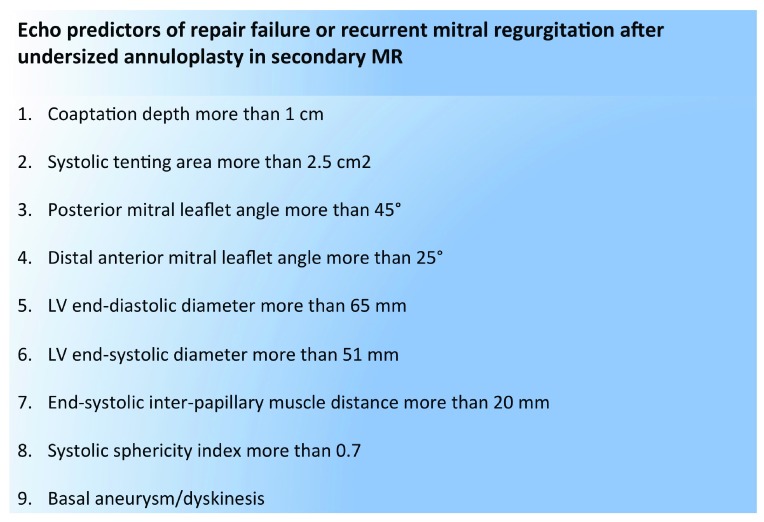
Echo predictors of repair failure or recurrent mitral regurgitation after undersized annuloplasty in secondary mitral regurgitation. LV, left ventricular; MR, mitral regurgitation.

Transesophageal echocardiography (TEE) is essential to understand MV anatomical morphology (leaflet, annular, and subvalvular anomalies), and it is fundamental to assess the degree of MR ( Table 1 and Figure 1), defining suitability for an optimal surgical or percutaneous MV repair ^10^. Moreover, the presence of thrombi in heart chambers or active endocarditic lesions, which could contraindicate intervention, should be detected. In particular for surgical patients with degenerative MR, both the leaflets and the corresponding associated lesions should be recognized and carefully studied. The management of asymptomatic patients is controversial as there are no randomized trials to support any particular course of action. Surgery can be proposed in selected asymptomatic patients with severe MR, in particular when repair probability is high. Some specific triggers for early intervention in these patients are worth mentioning: signs of LV dysfunction—in particular, LV ejection fraction (EF) of not more than 60% or LV end-systolic diameter (LVESD) of at least 45 mm or both—and lower LVESD are accepted in patients of small stature. If LV function is preserved, new-onset AF or pulmonary hypertension (at least 50 mmHg at rest or at least 60 mmHg during exercise) or both and sinus rhythm with severe LA dilatation (volume index of at least 60 ml/mq body surface area) provide surgical indication ^6^. The detection of annular calcification is a finding of paramount importance. Instead, for surgical patients presenting with secondary MR, echocardiographic parameters on the LV morphology and function (volume, EF, and sphericity index) are dominant in association with geometric MV distortion (tenting area, coaptation depth, leaflet angles, and inter-papillary muscle distance). Numerous predictors of MV repair failure after undersized annuloplasty have been identified ( Figure 2) and, when present, should suggest MV replacement as a more durable solution ^10^.

**Table 1.** Anatomical characteristics of both primary and secondary MR.

	Degenerat. Barlow	Degenerat. Fibroelastic Def.	Functional
**Age at Diagnosis**	< 50 years	> 50 years	N/A
**Valve Morphology** Annular dilatation Leaflets Chordae	(+++) Thickened (++) Excess tissue (+++) Heterogeneous Elongated	(+) Thin No excess tissue Thin(++) Ruptured	(++) Thickened (+) No excess tissue (++) Thin(+) Tethered
**Left ventricular** **dilatation**	N/A	N/A	(+++)

**Figure 3.**
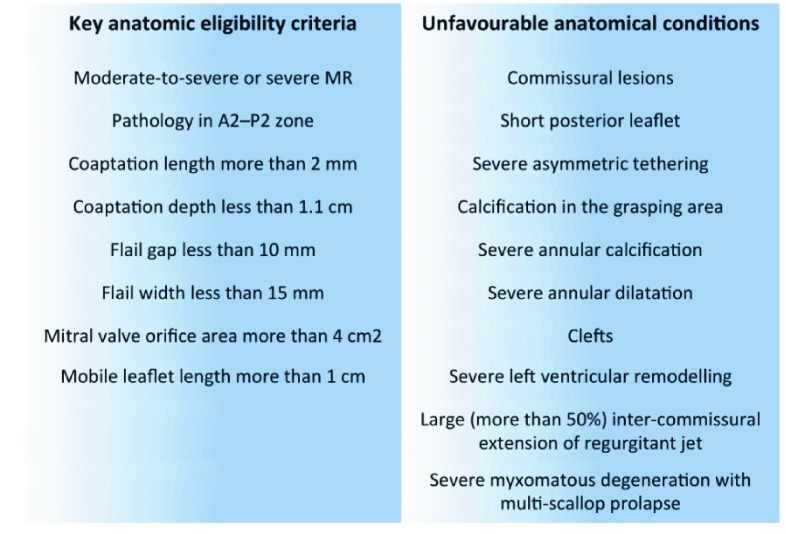
Key anatomic eligibility criteria for percutaneous edge-to-edge repair (EVEREST II) and unfavorable anatomical conditions. EVEREST II, Endovascular Valve Edge-to-Edge Repair Study; MR, mitral regurgitation.

TEE is mandatory in the operating room to confirm optimal competence of the valve after repair. Moreover, it is essential to confirm anatomical eligibility for percutaneous EE repair, where the anatomical criteria of the EVEREST II trial (Endovascular Valve Edge-to-Edge Repair Study) are the reference ( Figure 3). Percutaneous treatments beyond these criteria are now common, although certain anatomical conditions predict failure or suboptimal outcomes ( Figure 3).

## Degenerative mitral regurgitation

### Natural history, medical therapy, and timing of intervention

The natural history of severe degenerative MR is unfavorable and symptoms, age, AF, pulmonary hypertension, left atrial or LV dilatation, and low EF are all predictors of poor outcome ^5, 6^. There is no evidence-based medical therapy for patients with primary MR and minimal or no symptoms. Although beta-blockers and angiotensin-converting enzyme inhibitors palliate symptoms in patients with heart failure, they should not postpone the timing of intervention ^6^. Although patients with primary MR could remain asymptomatic for years, thus deferring the intervention, the treatment strategy has been redefined in recent years. Nowadays the international guidelines recommend earlier intervention when the probability of durable repair is high, especially when surgery can be performed by skilled teams with excellent outcomes. The purpose of ‘early repair’ is to treat severe degenerative MR before the occurrence of structural and functional changes in the left atrial and LV chambers, to ensure that survival and quality of life of patients are similar to those of the general population.

### Surgery

MV repair is the preferred surgical treatment for severe degenerative MR and has significant advantages over MV replacement ^6, 11^. The main goals (restitution of physiological leaflet motion, achievement of adequate leaflet coaptation, and annular stabilization with maintenance of an adequate mitral orifice) can be achieved by using a variety of isolated or combined techniques (leaflet resection, implantation of artificial chordae, chordal transposition/transfer, EE technique, and annuloplasty using a prosthetic ring or band) according to the type and location of the mitral lesions. Nowadays the vast majority of degenerative MR can be successfully repaired in dedicated valve centers ^12, 13^. Recent reports assess an absence from re-intervention at 10 years of more than 90%, slightly decreasing (to 80%) at 20 years ^14– 16^. Numerous anatomical lesions limiting long-term outcomes, especially the anterior or bileaflet prolapse, the extensive myxomatous disease, and annular calcifications, have been recognized so far. Surgical outcomes are strongly related to the experience of the center and surgeons. The hospital mortality achieved in dedicated centers is very low (less than 1%), and major complications are very rare when a strategy of early repair is adopted. Patients should be referred to centers with extensive experience to maximize the likelihood of a durable repair ^17, 18^. Indeed, the long-term outcomes are significantly improved when timely MV repair is performed, as opposed to lower late survival obtained in patients with hemodynamic decompensation (heart failure, depressed ventricular function, pulmonary hypertension, and arrhythmias) ^15, 19^.

### Percutaneous intervention

Several new transcatheter mitral devices are currently under investigation, although the MitraClip System (Abbott Vascular, Santa Clara, CA, USA), approved for use in high-risk or inoperable patients with severe MR and suitable anatomic criteria ( Figure 3), is the only one widely available, and around 30,000 implantations are performed worldwide ^20^. Percutaneous EE repair with this device is safe in degenerative MR and has low rates of procedural and 30-day mortality, complications (stroke, bleeding, tamponade, or resuscitation), and short mean hospital stay ^21– 23^. ****One-year survival is 80%, mirroring the advanced age and multiple comorbidities of the populations studied. Post-procedural mitral stenosis is very rare, and the clip detachment rate is around 2%. The acute procedural success rate (moderate or less than moderate final MR grade) is more than 80–85% and is maintained at 1- and 4-year follow-up. In the EVEREST II study ^21, 24^, which compared MV surgery versus transcatheter EE, 279 patients with moderate-to-severe and severe MR were randomly assigned to MitraClip repair (Abbott Laboratories, Abbott Park, IL, USA) or surgery (repair or replacement). The great majority of the population had degenerative MR and low risk of intervention. After 1 year, percutaneous repair was associated with a higher rate of residual MR, requiring surgical correction in comparison with surgery (20% versus 2%). These results were confirmed and remained stable after 4 years (25% versus 5%). Importantly, improvements in safety in favor of the percutaneous technique were influenced by the higher need for blood transfusion in the surgical arm. This population of the EVEREST II was significantly different from the patients who are currently treated in Europe, mainly affected by functional MR with severe LV dysfunction and higher surgical risk due to higher comorbidities. However, our group clearly documented that residual moderate MR after MitraClip implantation was associated with worse follow-up outcomes compared with mild or trivial MR, including survival, symptom relief, and MR recurrence. So far, better efficacy should be pursued by transcatheter mitral repair technologies in this particular setting ^25^. Accordingly, the study by Suri *et al*. demonstrated that also recurrent MR following surgical mitral repair in degenerative patients is associated with adverse LV remodeling and late death ^26^.

## Functional mitral regurgitation

### Medical therapy

Medical therapy (angiotensin-converting enzyme inhibitors, beta-blockers, and aldosterone antagonists) is mandatory in functional MR. Diuretics may be required for fluid overload, and vasodilators have a role in acute hemodynamic decompensation. The presence of conduction disturbances is not uncommon in these patients and contributes to the worsening of symptoms due to asynchronous ventricular contraction. Hence, cardiac resynchronization therapy aims at three different levels: (1) atrioventricular (2) intraventricular, and (3) interventricular. The therapy is achieved by pacing or sensing the right atrium, pacing the right ventricle (close to the septum), and pacing the left ventricle through the coronary sinus, also called biventricular pacing ^27^.

### Surgery

The best surgical treatment for secondary MR remains controversial ^27^. Mitral repair performed with an undersized rigid complete ring to restore leaflet coaptation and valve competence has been considered the standard treatment and can be performed with acceptable perioperative risk in carefully selected patients with secondary MR and poor LV function ^28, 29^. Several predictors of failure after repair have been recognized in the last decade, and it is well known that more advanced leaflet tethering predicts significant recurrent MR ^30– 37^. To improve MV repair durability, concomitant techniques on the subvalvular apparatus (secondary chordal resection, suturing of the posteromedial papillary muscle to the aorto-mitral continuity, infarct plication, papillary muscle imbrication, and posterior LV restoration) have been described in small, non-randomized, and observational studies and are under investigation ^30– 37^. Restrictive annuloplasty was recently compared with chordal-sparing MV replacement in a randomized study in patients with secondary MR of ischemic origin and demonstrated no advantage with regard to LV end-systolic volume index or 1-year mortality ^38^. However, the trial was underpowered for mortality and included patients with pre-operative predictors of repair failure in the repair group. A more appropriate selection of the candidates to mitral repair should be pursued since the rates of moderate-to-severe recurrent MR at 1 year were 32.6% in the repair and 2.3% in the replacement group. When the study follow-up was extended to 2 years, no significant difference between groups in LV reverse remodeling or survival was documented, and values of moderate-to-severe recurrent MR were 58.8% in the repair group and 3.8% in the replacement ^39^. Since no reverse LV remodeling was observed in the patients with recurrent MR, such a high rate of repair failure certainly had a major impact on the results. When repair was successful (no MR recurrence), the degree of LV reverse remodeling was higher than in patients submitted to MV replacement, emphasizing that a successful repair outplays the best MV replacement in this setting ^39^. Therefore, further studies are required to determine whether selected patients with secondary MR and no predictors of repair failure may benefit from surgical MV reconstruction. Moreover, no study has convincingly demonstrated a survival benefit compared with medical therapy in patients with MR and LV systolic dysfunction and this argues against surgical intervention in asymptomatic patients and poses a complex surgical decision in high-risk cases ^40^. However, in a large retrospective study recently published by Duke Medicine, substantial mortality was shown in patients with severe LV dysfunction and significant MR when treated with medical therapy alone whereas MV surgery was independently associated with higher event-free survival, encouraging the treatment of moderate and severe secondary MR in these challenging patients ^41^.

### Percutaneous intervention

Nowadays inoperable functional MR is widely treated with the MitraClip system, the transcatheter technology adopting the EE repair ^22, 23, 42– 44^. The outcomes of patients with functional MR and severe LV dysfunction in the ACCESS-EU (a prospective, multicenter, nonrandomized post-approval observational study of the MitraClip System in Europe) registry (around 400 patients) showed an extremely low mortality after 1 month (3%), which increased after 1 year to 17%, without evidence of significant complications (stroke, resuscitation, and tamponade) ^22, 23^. Significant residual MR in this challenging population, defined as moderate-to-severe or severe MR, progressively increased during the first year and was present in more than 20% of patients. With regard to clinical efficacy, most patients (70%) remained in New York Heart Association (NYHA) class I and II after the first year, showing either atrial or ventricular reverse remodeling, although in half of them a moderate residual MR was detected ^42– 46^. Direct comparisons between percutaneous EE repair and surgery in secondary MR are difficult since patients who receive either strategy are significantly different. Taramasso *et al*. reported in a non-randomized series higher efficacy of surgery compared with percutaneous intervention (freedom from moderate-to-severe and severe MR at 1 year was 94% versus 79%) ^43^. In contrast, *post hoc* analysis of the EVEREST II trial demonstrated equivalence of the two strategies in this setting ^24^. However, in the absence of a medical therapy control group, it is not possible to establish whether either treatment has a positive impact on survival; ongoing randomized studies will address this question. Currently, three large randomized trials will help clarify the future role of transcatheter devices in secondary MR therapy and whether MR reduction improves long-term outcomes: COAPT (Cardiovascular Outcomes Assessment of the MitraClip Percutaneous Therapy for Heart Failure Patients with Functional Mitral Regurgitation), RESHAPE-HF (Randomized Study of the MitraClip Device in Heart Failure Patients With Clinically Significant Functional Mitral Regurgitation), and MITRA-FR (Multicentre Study of Percutaneous Mitral Valve Repair MitraClip Device in Patients With Severe Secondary Mitral Regurgitation). The primary outcomes of these trials should be available at the end of 2017. In the COAPT trial (USA), 430 inoperable patients with secondary MR are randomly assigned between standard-of-care medical therapy and MitraClip versus standard-of-care medical therapy alone in order to assess the safety and effectiveness of the MitraClip in this field. In addition, the feasibility and safety of percutaneous direct mitral annuloplasty with Cardioband (Cardioband System; Valtech Cardio Ltd., Or Yehuda, Israel) have been recently assessed both in preclinical models and in humans ^47, 48^. The Cardioband system is a direct annuloplasty adjustable device that is implanted in the beating heart on the posterior annulus under fluoroscopic and TEE guidance. The human study group included 31 consecutive high-risk patients with moderate-to-severe or severe secondary MR ^48^. Procedural mortality was zero, and in-hospital mortality was 6.5% (2 of 31 patients, neither procedure- nor device-related). At 1 month, 88% of patients had moderate or less than moderate residual MR.

### Transcatheter mitral valve replacement early feasibility trials

Although this work aims to report the current management of MV repair, it is worth mentioning the current role of the complementary therapy: the transcatheter valve replacement. The feasibility of transcatheter MV replacement has been reported in a small number of patients at extreme risk (fewer than 100 patients) with native, MV disease but does not allow for any robust conclusions. On one hand, implantation of a valve in a non-calcified MV raises several important challenges: its positioning and anchoring, causing obstruction of the LV outflow tract, or coronary circumflex artery or paravalvular leak. On the other hand, transcatheter MV replacement has several theoretical advantages (compared with valve repair) because it is versatile and durably eliminates MR ^49^. Of the 10 ongoing studies, four are early feasibility trials in the US: Neovasc Tiara Mitral Valve System (TIARA-I; NCT02276547), Tendyne Mitral Valve System (NCT02321514), CardiAQ TMVI System (Transfemoral and Transapical DS; NCT02515539), and Twelve Transcatheter Mitral Valve Replacement (NCT02428010).

## Conclusions

Nowadays surgical MV repair is considered the gold standard for patients with severe degenerative MR. To ensure the best durable outcomes, the procedure should be performed in a timely manner and in dedicated centers. In patients with secondary MR and dilated cardiomyopathy, the mitral repair intervention is more challenging and the careful selection of patients is essential. The presence of echocardiographic predictors of postoperative residual or recurrent MR should be carefully considered to recommend replacement as a more durable solution. Percutaneous interventions offer beating-heart MV repair and replacement under physiological conditions, without the need for cardiopulmonary bypass. Beyond percutaneous EE repair, the percutaneous direct annuloplasty reproduces proven surgical techniques, showing a good safety profile and efficacy. Advanced imaging technologies (three-dimensional echocardiography and heart computed tomography scan) will guide MV repair procedures in the near future.
